# South African Thoracic Society statement on obstructive airways disease and COVID-19

**DOI:** 10.7196/AJTCCM.2020.v26i3.111

**Published:** 2020-10-13

**Authors:** R van Zyl-Smit, C Feldman, G Richards, S Abdool-Gaffar, U Lalloo, I Kalla, C F N Koegelenberg, K Dheda

**Affiliations:** 1 Division of Pulmonology, Department of Medicine and UCT Lung Institute, Faculty of Health Sciences, University of Cape Town, South Africa; 2 Faculty of Health Sciences, University of the Witwatersrand, Johannesburg, South Africa; 3 Kingsway Hospital, Amanzimtoti, South Africa; 4 Durban University of Technology, Enhancing Care Foundation and Busamed Gateway Private Hospital, Durban, South Africa; 5 Division of Pulmonology, Faculty of Health Sciences, Stellenbosch University and Tygerberg Hospital, Cape Town, South Africa; 6 Centre for Lung Infection and Immunity, Division of Pulmonology, Department of Medicine and UCT Lung Institute, University of Cape Town, South Africa; 7 South African MRC/UCT Centre for the Study of Antimicrobial Resistance, University of Cape Town, South Africa; 8 Department of Infection Biology, Faculty of Infectious and Tropical Diseases, London School of Hygiene and Tropical Medicine, London, UK

## Abstract

- Asthmatics do not appear to have increased susceptibility to COVID-19.
- Uncontrolled severe asthma may be associated with worsened COVID-19 outcomes, especially in asthmatics managed with oral corticosteroids.
- Risk mitigation measures such as hand hygiene, social distancing and wearing of face masks must be observed at all times.
- Asthma should be managed as outlined in local and international guidelines.
- Ensure an adequate supply of medication, and inhaled corticosteroids should not be withdrawn
- Chronic obstructive pulmonary disease (COPD) is associated with severe COVID-19 disease and poor outcomes.
- Maintenance of background medication is important to avoid exacerbations of COPD.
- Vaccination against influenza is strongly advised for all patients with asthma and COPD
- Vaccination against pneumococcal infection is advisable for patients with COPD.
- Patients with obstructive airway disease on oral corticosteroids and/or with impaired lung function should take stringent safety precautions.

This statement will be updated when more data become available

Asthma and COPD occur commonly in South Africa. SARS-CoV-2 is a novel coronavirus, which can result in COVID-19-associated severe respiratory infection with respiratory failure and the need for mechanical ventilation. The South African Thoracic Society has prepared a guidance statement to assist clinicians and patients with asthma and COPD during the current epidemic.

## Asthma

Asthmatics do not appear to have increased susceptibility to COVID-19.^[1]^ However, when asthmatics contract COVID-19, they may have a worse outcome. Thus, while asthma was not documented as a risk factor/ underlying comorbidity for COVID-19 infection in earlier studies, some more recent studies, particularly from the USA, have shown that asthma may be a significant comorbid condition.^[2,3]^ Furthermore, asthma was also found to be relatively common among intensive care unit cases with COVID-19 and in patients who died. Therefore, while mild to moderate asthma that is well controlled may not be a risk factor for COVID-19, people with uncontrolled and severe asthma, those receiving repeated or regular doses of oral corticosteroids (OCS) and those with underlying structural lung damage may be at risk of more severe COVID-19.

Recently published data from the UK primary care and COVID-19 system notification databases have indicated that asthma (without corticosteroid use) was not associated with higher mortality when adjusting for confounders in 10 926 COVID-19-associated deaths in the UK.^[4]^ Asthma with recent use of OCS was associated with higher mortality compared with non-asthmatics when fully adjusted for other variables (hazard ratio (95% confidence interval) 1.13 (1.01 - 1.26)).^[4]^ By contrast, the International Severe Acute Respiratory and Emerging Infection Consortium (ISARIC) study reported that 14% of hospitalised patients with asthma have no increased risk of death.^[5]^ Data from Spain^[3]^ suggest that a few patients with asthma were infected with SARS-CoV-2 and this was not associated with asthma exacerbation

Regarding other asthma phenotypes and patients from other settings, there is little data available, and thus recommendations cannot be made. Few asthma patients are recorded in published cohorts of SARS-CoV-2 infections in China, and it has not been linked to poor outcomes.^[6,7]^ Although data from the previous severe acute respiratory syndrome and Middle East respiratory syndrome outbreaks did not demonstrate any increased risk of infection nor severe disease in asthmatics, this may be different for COVID-19, and further data are awaited.^[8–12]^ Data from previous influenza outbreaks and the 2009 H1N1 pandemic suggest that asthma was associated with hospitalisation,^[13]^ but interestingly, protective for mortality.^[14]^

Based on these limited data, severe asthmatics (especially if receiving OCS) seem to be at higher risk of death than non-asthmatics. There is no evidence to suggest that inhaled steroids should be stopped as they are protective against asthma exacerbations.^[15]^ Inhaled or OCS should not be withheld if indicated to treat exacerbations of asthma.

## Chronic obstructive pulmonary disease

Current evidence summarised in two meta-analyses indicate that chronic obstructive pulmonary disease (COPD) patients are at a significantly higher risk of severe COVID-19 and adverse outcomes. Lippi *et al*. ^[14]^ reported on 1 592 patients and demonstrated a significant association between COPD and poor outcomes in COVID-19 (odds ratio 5.69 (2.49 - 13.00)). The second meta-analysis included 15 studies with 2 473 patients. The patients with COPD had a relative risk of 1.88 (1.4 - 2.4) for severe COVID-19 compared with non-COPD patients.^[16]^ These data provide sufficient evidence establishing an association between COPD and risk of severe COVID-19. Tobacco smoking has also been associated with severe COVID-19 and adverse outcomes.^[17]^ It is unclear, however, if smoking is an additive risk factor in addition to COPD. It is worth noting that data are discordant and in both the UK^[17]^ and China,^[18]^ COPD did appear to confer increased risk of death among hospitalised and confirmed cases, respectively.^[18,19]^

Influenza and pneumococcal vaccination are recommended as well as a regular supply of background medication.^[20]^ Inhaled steroids are a risk factor for bacterial pneumonia and should be avoided unless indicated^[19]^ and where dual bronchodilators are not accessible.
